# Exposure to animal suffering, adult attachment styles, and professional quality of life in a sample of Italian veterinarians

**DOI:** 10.1371/journal.pone.0237991

**Published:** 2020-08-27

**Authors:** Alessandro Musetti, Alessandro Schianchi, Luca Caricati, Tommaso Manari, Adriano Schimmenti

**Affiliations:** 1 Department of Humanities, Social Sciences and Cultural Industries, University of Parma, Parma, Italy; 2 Private Practice, Fornovo di Taro, Parma, Italy; 3 Faculty of Human and Social Sciences, UKE - Kore University of Enna, Cittadella Universitaria, Enna, Italy; Fukushima Medical University School of Medicine, JAPAN

## Abstract

Contextual and individual risk factors of veterinarians’ professional quality of life are being debated. Research suggests that attachment styles are relevant predictors of professional quality of life; however, their role in work-related well-being of veterinarians is yet to be ascertained. In the present study, self-report measures on exposure to animal suffering, adult attachment styles, and professional quality of life were administered to 1,445 Italian veterinarians (70% females) aged 24 to 74 years old; sociodemographic information and information on workload were also collected. Female gender, higher levels of ordinary workload, on-call hours per week, exposure to animal suffering, together with fearful and preoccupied attachment styles were significantly associated with lower levels of veterinarians’ quality of life. This suggests that work-related factors may combine with individual psychological features in promoting or disadvantaging the professional quality of life of veterinarians. Implications of these findings for promoting veterinarians’ quality of life and directions for future research are discussed.

## Introduction

Health-care providers’ well-being and mental health have received an increasing amount of attention in the last few decades [1–4]. Evidence indicates that different categories of human-care professionals (e.g., physicians and nurses) are at risk for stress-related pathology and poor well-being [5, 6]. More recently, animal-care providers (e.g., veterinarians and veterinary nurses) also have been identified as professionals who might suffer from high levels of occupational stress (e.g., burnout, [7–9]) and moderate-to-severe patterns of psychopathological symptoms [10, 11], including substance use [12], depression [13–15], and suicidal ideation [16–21].

In fact, in the course of their work, veterinarians are exposed to a wide variety of stressful situations as a direct result of their complicated role as health-care providers for animals and as helpers for the worried or even grieving owners [22, 23]. In fact, research has suggested that the emotional burden experienced by animal care providers may be similar or even greater than that experienced by their human care counterparts [23]. Nevertheless, there is no definitive evidence on this issue.

The long-term effect of occupational stressful factors on veterinarians’ well-being has been described in a range of prior literature, in terms of secondary traumatic stress (e.g., [24]), secondary victimization (e.g., [25]), vicarious traumatization (e.g., [26]), moral distress (e.g., [27]), burnout (e.g., [28]), and compassion fatigue (e.g., [29]). While each of these constructs has specific theoretical and clinical implications, they all encompass the experiences of professionals dealing with clients’ suffering or emotional distress. According to Tekippe and Krejci [30], veterinarians frequently undergo an “emotional rollercoaster” because daily they deal with varied kinds and severities of animal clinical conditions, combined with equally varied emotional expressions on the part of animal owners (e.g., an owner’s mourning for his or her euthanized pet followed by an owner’s elation over his or her healthful new puppy).

Therefore, professionals working in animal welfare and animal care, such as veterinarians, veterinary nurses, and animal shelter workers, may experience moral stress as a result of being involved in morally sensitive activities, such as caring for sick, injured, or dying animals; performing euthanasia; and exposure to animal cruelty or abuse [31–33]. Unless properly addressed, fatigue and chronic stress are experienced and may lead to a deep state of exhaustion and physiological and emotional impairment [34–36], which might exacerbate ongoing stress-related symptoms and might even lead to suicide [7, 8, 16, 29, 37]. Moreover, occupational stressors associated with clinical work with animals include long working hours, client expectations, unexpected outcomes, communicating bad news, poor work–life balance, high workloads, rising veterinary care costs, and professional isolation [18, 29, 38–40]. Numerous studies have observed that young female veterinarians have a higher risk of developing mental health problems and are more likely to consider suicide, compared to their male colleagues and senior veterinarians (e.g., [41].

Despite these negative effects, trying to improve the health of animals and helping others do bring joy and a sense of professional achievement [22, 42–44]. Volk and colleagues [45] evaluated a large group of American veterinarians and found significant variations in well-being levels (i.e., satisfaction with professional life) depending on their gender and age. On average, male practitioners had higher scores on well-being than their female colleagues, and married veterinarians scored higher than those who were single or divorced. As a group, older veterinarians scored higher than younger veterinarians did, and owners scored higher than associates did. Professional factors most associated with high levels of well-being were higher income, fewer working hours, low or no student debt, practice ownership, and evenings off work.

Because of the multidimensional complexity of the topic, it is important to take into account the broad extent of professional quality of life when considering trauma work, such as working with suffering animals. To this end, a set of three factors—compassion fatigue, burnout, and compassion satisfaction—has been posited to account for such variability in professional quality of life [46–49]. Compassion fatigue describes the “cost of caring” [50 p.1] that health-care providers may experience by supporting trauma victims. Specifically, it is defined as a deep emotional and physical exhaustion that can affect health-care providers over time because of prolonged exposure to patients’ suffering, and it may evoke detrimental effects on coping resources and a decay in work performance [51–53]. Burnout is a form of physical and emotional exhaustion associated with long-term involvement in work-demanding situations (e.g., excessive workload or unfavorable work conditions) [50]. Burnout differs from compassion fatigue in that the former is directly associated with the number of daily stressors and hassles occurring in the workplace [54], whereas the latter is a possible negative outcome of being exposed to a patient’s trauma [55]. Lastly, compassion satisfaction refers to the positive side of caring [56] and involves feelings of satisfaction and fulfillment related with helping those who suffer [49, 57]. Health-care providers who experience compassion satisfaction report high levels of job commitment, job satisfaction, and job performance [49, 58]. Thus, professional quality of life is the experience of well-being associated with the overall balance between work-related distress and satisfaction.

The scientific literature has explored extensively the trio of factors consisting of compassion fatigue, burnout, and compassion satisfaction among many categories of health-care providers, including physicians, nurses, and trauma therapists [59–62]. By comparison, there are fewer studies dealing with professional quality of life in veterinarians [24], and to the best of our knowledge, no studies have been conducted on Italian veterinarians to date. Furthermore, a certain number of studies gave support to some contextual sources of stress, such as long working hours or being on call [63–65], showing higher levels of veterinarians’ compassion fatigue and burnout in comparison with the general population (e.g., [29, 42, 45]). However, a limited consensus has been reached within the literature about the role of other factors, such as individual psychological variables, which may foster or restrain professional quality of life among health-care providers in general and among veterinarians specifically [24]. Nevertheless, within the same organization and occupation, some professionals show a low professional quality of life, whereas others do not, suggesting that individual psychological variables might play a key role. In this vein, personality characteristics have been linked to compassion fatigue [66, 67], burnout [68, 69], and compassion satisfaction [70, 71]. To date, a significant gap remains in our understanding of the underlying dynamics and the specific internal factors that contribute to variability in health-care providers’ responses to exposure to human or animal suffering.

Attachment theory is a sound theoretical framework to account for the complexity and multi-determined nature of the contextual factors and the psychological processes associated with professional quality of life, which originally was formulated by Bowlby [72, 73] and subsequently is undergoing a vast expansion of its original scientific foundations alongside its educational and treatment implications. Attachment theory highlights the function of early parental bonding for regulating emotional distress, fostering safety, and promoting security in life and relationships [74, 75]. For example, when a distressing event occurs, a securely attached child is sustained emotionally by the caregiver who is responsive to his or her needs, and ultimately, the child learns to deal with stressful situations as they arise. During adulthood, the internal representations of these positive childhood experiences provide a frame of reference for developing adequate relational bonds with others, for regulating emotions, and for coping with distressing life events. Conversely, if attachment relationships in childhood are characterized by negative experiences, such as continuous failures in communication, neglect, or abuse, the child will develop insecure internal working models of attachment [73] that will lead him or her to excessive anxiety and/or avoidance of close and intimate relationships in later life stages [76], which might foster a number of difficulties in emotional adjustment [74, 77–79]. Therefore, attachment styles are trait-like factors [80]. However, though relatively stable over time, their manifestations are modulated by life events and the context of current relationships [81, 82]. In fact, attachment behaviors are modeled onto the internal representations of self and others deriving from childhood experiences but manifest themselves differently within specific relationships [83]. Hence, this construct may be relevant for understanding the use of specific coping strategies to deal with psychological and physical pain among care providers and may further advance knowledge in the field beyond already well-studied and highly stable dispositional factors, such as temperament [84].

Furthermore, attachment theory provides a useful and well-documented framework in the context of human–animal interactions ([85, 86]. In this regard, Beck and Madresh [87] provided evidence that human–companion animal and human–human relationships shared similar attachment structures (representations of self and others) and styles (secure or insecure).

As for the regulatory nature of attachment, Mikulincer [88] posited that securely attached individuals use constructive and effective coping strategies to deal with stress because of their positive representations of their own abilities and of others’ responsiveness to their needs. Furthermore, secure attachment is predictive and significantly associated with the ability to cope successfully with compassion fatigue, thus mitigating and minimizing vulnerability to its negative effects [89–91]. In addition, a large body of empirical and theoretical literature has highlighted the link between insecure attachment styles (high levels of anxiety and/or avoidance) and various forms of psychopathology [92–94], including post-traumatic stress [95]. Accordingly, insecure attachment styles in adulthood, such as dismissive, preoccupied, and fearful attachment styles, consistently have been associated with increased symptoms of emotional distress, maladaptive use of psychological defenses, and cognitive distortions [76].

Differences among attachment styles and work-related feelings have been investigated in the literature. For example, Hazan and Shaver [96] found that securely attached workers reported high levels of work satisfaction and an overall well-being; in sharp contrast, workers with anxious attachment reported feelings of job insecurity, fear of rejection, poor performance, and lack of appreciation and recognition by coworkers. Whereas avoidant attached workers reported dissatisfaction with coworkers despite maintaining their satisfaction with job security.

However, more recently researchers have begun to take into account how attachment styles may be relevant in other domains, including professional quality of life of health-care providers [97]. Previous studies have shown that when an insecure-attached health-care provider is exposed to others’ cumulative suffering, he or she is more prone to experience higher levels of distress, burnout (e.g., [98–100], see [97] for a review), and compassion fatigue (e.g., [101]; see [97] for a review) and higher levels of compassion satisfaction (e.g., [102]) than secure attached colleagues. Considering that attachment styles are relevant for understanding professional quality of life, and that they also play a role in the human–animal interactions, it may be relevant to fill a gap in the literature concerning the lack of research on the relationships between professional quality of life and attachment styles among veterinarians.

Referring to the attachment theory and based on previous empirical studies on the relationship between attachment styles and quality of life in other professions, in the present study, we aimed to investigate the relationships between exposure to animal suffering, attachment styles, and professional quality of life dimensions in Italian veterinarians. To the best of our knowledge, this is the first study addressing the relationship between these variables in an Italian sample.

Accordingly, this study examined how the contextual factors related to the work of veterinarians (e.g., the level of exposure to animal suffering) and the individual factors related to the security of approach to life and relationship (e.g., attachment styles that play a key role in regulating emotions evoked by stressful events) contributed to the professional quality of life among the Italian veterinarians. Based on theoretical considerations and previous findings, we specifically expected that increased hours of work per week, on call availability, exposure to animal suffering, and insecure attachment styles would positively predict compassion fatigue and burnout and would negatively predict compassion satisfaction.

## Methods

### Data collection and ethic concerns

The study adopted a cross-sectional research design involving an anonymous online self-report survey. We invited licensed veterinarians to participate in the present research with an e-mail sent by the Federation of Italian Professional Veterinary Associations (ANMVI), which is the largest and most representative organization of veterinarians in Italy. In July 2019, an e-mail was sent to all 17,400 potential participants illustrating the purposes of the research, indicating who the addressee of the research was, and specifying the anonymous and voluntary nature of the present study. Data were collected between July 29, 2019, and October 2, 2019. Participants provided written online consent and completed all measures using Survey Monkey (www.surveymonkey.com). Potential participants were informed of the voluntary nature of participation in the study and of the anonymity of their responses (no data on the responders’ identifications were collected, including their Internet protocol address). The study was designed and carried out according to the Ethical Code of the Italian Association of Psychology, the European Code of Conduct for Research Integrity, and the American Psychological Association.

### Measures

#### Demographic questionnaire

Participants completed a demographic questionnaire that assessed age, gender, marital status, working status, working sector, average number of hours worked per week, and work on call availability (yes/no).

#### Professional quality of life

An adapted version for veterinarians of The Professional Quality of Life Scale (ProQOL) developed by Stamm [103] and validated in Italy by Palestini, Prati, Pietrantoni, and Cicognani [104] was administered. It presented 22 items rated on a 1 (*never*) to 5 (*very often*) scale to obtain three measures. (a) Compassion satisfaction (CS; eight items; e.g., “My work makes me feel satisfied”) reflected well the pleasure derived from being able to care for others. (b) Burnout (BO; seven items; e.g., “I feel overwhelmed by the amount of work or the size of my caseload I have to deal with”) consisted of a state of psychophysical exhaustion, particularly associated with feelings of hopelessness and with difficulties to deal with one’s work effectively in the long-term. (c) Compassion fatigue (CF; seven items; e.g., “I jump or am startled by unexpected sounds”), was defined as a state of tension and preoccupation associated with a reduction of empathizing, nurturing abilities, and emotional coping resources in caregivers who worked with animal patients and human clients. The ProQOL thus reflected on the positive (CS) and negative (BO, CF) aspects of veterinarians’ practices. The sum of the scores in each subscale determined the level of CS, BO, and CF experienced by an individual.

#### Attachment styles

The Italian translation of the Relationship Questionnaire (RQ; [105]; Italian adaptation by Carli, [106]) was used to assess attachment styles. Starting from the assumption that attachment styles entail views of one’s own self-worth and of the trustworthiness of others, positive versus negative view of self, and positive versus negative view of other dimensions were combined to create four different prototypical attachment attitudes: (a) secure (“I feel comfortable depending on others and having others depend on me,” entailing a positive view of self and others); (b) dismissing (“I am comfortable without close emotional relationships,” entailing a positive view of self and a negative view of others); (c) preoccupied (“I want to be intimate with others, but I often find that others are reluctant to get as close as I would like,” entailing a positive view of others and a negative view of self); and (d) fearful (“I find it difficult to trust others completely, or to depend on them,” entailing a negative view of self and others). The participants were required to indicate on a 7-point Likert scale how well each paragraph described them from 1 (*It does not describe me at all*) to 7 (*It very much describes me*). Higher scores on each item reflected greater levels of that attachment style. RQ is a widely used [107] and well-validated tool of adult attachment styles [108].

#### Exposure to animal suffering

Participants completed four ad hoc items that assessed their exposure to the pain, anguish, and death of animal patients in a typical month of the past year (e.g., “How many times have you seen animals in situations of serious suffering”). These items were developed based on previous literature [109] and were revised through pilot testing on a panel of veterinarians.

### Plan of the analysis

Zero-order correlations were inspected preliminarily to detect potential problems about collinearity and discriminant validity. Subsequently, three regression models were tested using a structural equation modelling (SEM) framework and considering compassion satisfaction, burnout, and compassion fatigue as dependent factors. We choose to use SEM as it permits taking into account several dependent factors at a time. This produces estimates that are controlled for the effects of other outcomes, thus limiting the number of separate tests and reducing the likelihood of type-1 error. Demographics, suffering exposure, and types of attachment were added as predictors in all the models to make them comparable. However, model 1 tested the effect of demographics by forcing other estimates to be zero. Model 2 estimated the effects of demographics and suffering exposure but forced the effects of attachment to zero. Finally, in model 3, all the effects were allowed to be estimated freely. In this way, model 1 was nested in model 2 that, in turn, was nested in model 3. To compare the models, we considered the values of Akaike’s information criterion (AIC) and Bayesian information criterion (BIC; for AIC and BIC, lower values indicate a better model; if BIC and AIC suppled discordant information, we decided to consider primarily BIC, as it is more penalizing than AIC), as well as R-squared values for each outcome variable. SEM was estimated using maximum likelihood and robust standard error estimation, which is robust to the violation of normality assumption.

## Results

### Sample socio-demographics

Data from 1,904 respondents were collected with a response rate of 10.94%. Of these, 434 missed one or more scales and were excluded, and a further 25 participants reported values out of the range of the scale on the attachment questionnaire. The final database consisted of 1,445 participants. The veterinarians who participated in this research were aged between 24 to 74 years (*M* = 43.27, *SD* = 11.10) and were predominantly female (*n* = 1011; 70.00%); trends show that the number of females is still increasing consistently with the Italian population of veterinarians [110 p. 17]. S1 Table includes the sex differences for continuous variables. The majority of respondents reported companion animal practice as their main type of work (*n* = 1227; 84.90%). The majority of respondents were also employed full-time (*n* = 1134, 78.5%), with a median of 40 hours of work per week (*M* = 40.54, *SD* = 24.07) and 12 hours of on-call (*M* = 24.07, *SD* = 29.23). Table 1 indicates the sociodemographic and occupational characteristics of the sample.

**Table 1:** Socio-demographic and occupational characteristics of the participant.

	N = 1445
**Gender**	
Males	434 (30.0%)
Females	1011 (70.0%)
**Marital status**	
Married/domestic partner	1024 (70.9%)
Single	326 (22.6%)
Divorced/separated	86 (6.0%)
Widow	9 (0.6%)
**Working status**	
Full-time employed	1134 (78.5%)
Part-time employed	187 (12.9%)
Casual workers	85 (5.9%)
Unemployed	0 (0.0%)
Missing	39 (2.7%)
**Working sector**	
Companion animal practice	1227 (84.9%)
Production animals	69 (4.8%)
Pharmaceutical / feed company	15 (1.0%)
University	23 (1.6%)
Veterinary local units	49 (3.4%)
Other	62 (4.3%)

### Preliminary analysis

Table 2 shows zero-order correlations and descriptive statistics of considered variables. As indicated, correlations were generally low to moderate in magnitude, so that no concerns about collinearity or discriminant validity appeared.

**Table 2:** Descriptive statistics, zero-order correlations, and Cronbach’s alphas.

	1	2	3	4	5	6	7	8	9	10	11	12
1. Compassion Satisfaction (α = 0.88)	1											
2. Burnout (α = 0.88)	-0.36**	1										
3. Compassion Fatigue (α = 0.84)	-0.23**	0.55**	1									
4. Gender	0.09**	-0.17**	-0.22**	1								
5. Age	0.20**	-0.17**	-0.02	0.26**	1							
6. Hours of work per week	0.07*	0.20**	-0.02	0.16**	-0.21**	1						
7. On call (yes/no)	0.13**	0.08**	0.07*	0.08*	0.16**	0.11**	1					
8. Exposure to animal suffering (α = 0.80)	0.00	0.16**	0.10**	0.08*	-0.14**	0.20**	0.06*	1				
9. Secure attachment	0.20**	-0.16**	-0.15**	0.05	0.04	-0.01	-0.01	0.00	1			
10. Fearful attachment	-0.23**	0.27**	0.28**	-0.17**	-0.23**	0.03	-0.00	0.04	-0.25**	1		
11. Preoccupied attachment	-0.15**	0.20**	0.19**	-0.06*	-0.16**	0.00	-0.04	0.07*	-0.08**	0.28**	1	
12. Dismissing attachment	0.00	-0.03	-0.06*	0.08*	0.05^	0.03	0.03	0.02	-0.36**	-0.12**	-0.20**	1
*M*	28.10	23.99	13.80	0.30	43.27	40.54	0.66	15.55	3.73	2.84	2.26	3.82
*SD*	5.22	5.78	4.97	0.46	11.10	14.32	0.47	11.81	2.06	1.94	1.69	2.22

* *p* < .05;

** *p* < .01.

### Testing the models

Table 3 shows the SEM results for the all three tested models. As indicated, female participants were more likely to report burnout and compassion fatigue than male participants were, while no sex differences appeared for compassion satisfaction. Age of participants and workload (i.e., worked hours per week) were positively associated with compassion satisfaction and burnout, but not with compassion fatigue. On call availability was associated positively with compassion fatigue, compassion satisfaction, and burnout. Demographics explained about 6% of variance for compassion satisfaction and compassion fatigue and about 10% of the variance for burnout. Adding suffering exposure in model 2 did not change estimates of demographics and did not improve substantially the variance explanation of outcomes. Accordingly, model 2 had a slightly better fit than model 1. In any case, the effect of suffering exposure appeared to be significant on burnout and compassion fatigue, while it had no significant effect on compassion satisfaction. Finally, in model 3, secure attachment was positively linked to compassion satisfaction and negatively linked to burnout and compassion fatigue. Inversely, fearful and preoccupied attachment styles were negatively associated with compassion satisfaction and positively linked with burnout and compassion fatigue. Dismissing attachment appeared to be unrelated with dimensions of professional quality of life. It is worth noting that adding types of attachments in the model change only the effect of age of participants on burnout and compassion fatigue. More precisely, the participant’s age lost its association with burnout and became significantly linked to compassion fatigue when attachments were entered in the equation. Overall, model 3 substantially increased the explained variance for all dimensions of professional quality of life, as variance explained for compassion satisfaction, burnout, and compassion fatigue was about 12%, 18%, and 16%, respectively. Accordingly, the AIC and BIC of model 3 were markedly lower than those of model 1 and 2, so that model 3 appeared to be better than other models in explaining variations in the quality of life dimensions.

**Table 3:** SEM results.

	Model 1			Model 2			Model 3		
	CS	BO	CF	CS	BO	CF	CS	BO	CF
Demographic and work-related factors									
Gender (0 = woman)	0.017	-0.187***	-0.240***	0.017	-0.198***	-0.251***	-0.008	-0.169***	-0.219***
Age	0.200***	-0.095**	0.034	0.200***	-0.077**	0.051	0.155***	-0.018	0.117***
Workload	0.102**	0.198***	0.014	0.102**	0.178***	-0.006	0.100**	0.183***	0.000
On call (0 = no)	0.081**	0.090**	0.085**	0.081**	0.083**	0.078**	0.089**	0.076**	0.070**
Contextual factor									
Exposure to animal suffering				0.002	0.129***	0.129***	0.008	0.119***	0.118***
Psychological factors									
Secure attachment							0.164***	-0.109***	-0.096**
Fearful attachment							-0.137***	0.170***	0.202***
Preoccupied attachment							-0.067**	0.121***	0.122***
Dismissing attachment							0.011	-0.026	-0.042
R^2^	0.058	0.095	0.059	0.058	0.110	0.074	0.123	0.184	0.161
ΔR^2^	0	0	0	0	0.015	0.015	0.065	0.074	0.087
AIC (ΔAIC)	25762.177			25731.118 (-31.059)			25533.716 (-197.402)		
BIC (ΔBIC)	25872.970			25857.739 (-15.231)			25723.647 (-134.092)		

*N* = 1,445;

** *p* < .01;

*** *p* < .001.

Standardised coefficients are reported.

## Discussion

This study aimed to extend the limited literature concerning professional quality of life in veterinarians by applying the theoretical framework of attachment theory. The results of this study generally confirmed our hypothesis that each category of predictors (i.e., demographic, work-related, contextual, and psychological factors) were associated with the trio of professional quality of life factors: compassion fatigue, burnout, and compassion satisfaction. Although not directly comparable, present results about ProQoL are consistent with previous results indicating that veterinarians score higher on compassion satisfaction than on dimensions linking to compassion fatigue [29, 109, 111]. As concerns demographic variables, according to other studies [10, 15], SEM results showed that females reported a higher risk of experiencing negative outcomes, such as compassion fatigue and burnout. Reijula and colleagues [64] highlighted that female veterinarians could feel they were highly pressed for time and could have difficulty reconciling work with their private lives (e.g., in the case of pregnancy). Nonetheless, further studies are required to investigate the potentially different sources of distress between male and female veterinarians in more depth. As for the veterinarians’ age, younger professionals showed more psychological distress and less compassion satisfaction, but when attachment styles were entered in the model, the age of participants was no longer significantly associated with burnout. This might have occurred because the participants’ ages were associated negatively with fearful and preoccupied attachment styles, so that entering attachments in the regression could have suppressed the effect of age on burnout. This finding, in line with the literature [64], also may be due to lower social recognition and lower incomes of younger veterinarians than their older colleagues [45] or, as an alternative interpretation, may depend on the enhancement of coping skills gained with work and life experiences [10], [18]. Consequently, strategies specifically planned to support younger veterinarians in managing work-related stress might be needed and might be effective in improving young veterinarians’ job satisfaction.

Unsurprisingly, our results confirm that work-related factors, such as workload and on call availability, were associated with veterinarians’ work-related stress (e.g., [18, 112]). Less obvious was the positive association between the workload and compassion satisfaction; however, as Stamm [49] stressed, a health-care provider can be compassion-fatigued from his or her work while experiencing the positive benefits from it.

Our findings support that the “cost of caring” [50 p. 1] is also a relevant issue in the field of animal health-care professionals. Regarding the contextual factors of animal care providers’ settings, the findings indicated that exposure to animal suffering was associated with compassion fatigue and burnout. This may be because a veterinarian, who is exposed to a high level of animal suffering (i.e., euthanasia administrations), may also incur other work-related stressful events (i.e., a lack of emotional support from colleagues; see [33]). Moreover, although these two components of professional quality of life are conceptually different, as in previous empirical studies [113], we found a positive correlation between compassion fatigue and burnout. In this vein, it has been noted [114] that these constructs are closely related but different and sometimes ambiguously defined. Further research is warranted to elucidate the relationship between burnout and compassion fatigue in veterinarians.

Finally, the last objective of the study was to evaluate the association between attachment styles and veterinarians’ professional quality of life dimensions. Our findings concur with previous studies showing that health-care providers tend to respond to work-related stressful events through an individual lens affected by their own attachment styles [97]. Secure attachment style (low anxiety, low avoidance) was predictive of veterinarians’ overall higher levels of professional quality of life, suggesting that it may serve as an internal resource that enhances work adjustment and well-being by sustaining a more effective coping strategy for emotional distress [90, 115]. Further, our findings support the hypothesis that preoccupied (low avoidance, high anxiety) and fearful (high avoidance, high anxiety) insecure attachment styles were associated positively with compassion fatigue and burnout and associated negatively (albeit weakly) with compassion satisfaction. These findings are consistent with the view that insecure attachment styles, comprised of a negative view of the self, are associated with intensified negative emotional responses [116]. A dismissing attachment style (high avoidance, low anxiety) was not associated with any of the professional quality of life dimensions. It is interesting to note here that individuals with high levels of attachment anxiety nurture doubts regarding their self-worth, concerns about attachment figures not being available at times of need, hypervigilance, and an increased appraisal of threat [117]. Whereas, individuals with high levels of attachment avoidance tend to keep a behavioral distance and emotional independence from others because of their negative representations of others [118, 119]. Hence, the latter may feel less emotionally involved in their role as helpers or may be uninterested in support from colleagues when facing adverse events. Overall, the predictive effects of attachment styles on veterinarians’ professional quality of life may suggest that the attachment dimensions are involved in their psychological approaches to their activities, their patients, and their work in general.

Looking at our results as a whole, it can be seen that some of the evaluated dimensions (e.g., high-anxiety attachment styles and exposure to animal suffering) may play a specific role in predicting veterinarians’ professional quality of life levels. Whereas, others might only be indicators of increased risk shared with non-health-care professionals (e.g., work overload [120]) or the general population (e.g., female gender [121]).

As with all research, the present study comes with a number of limitations. The investigation used a cross-sectional and correlational design so it was not possible to establish a cause-effect relationship between the evaluated variables. In fact, the structural model of the relationship among the investigated variables were invoked based on theory. Thus, even though the statistical testing has provided significant and theoretically consistent results, our study cannot entirely rule out the presence of a different structure of relationships among the investigated variables. In addition, the cross-sectional design may have introduced bias, given the common method variance and social desirability [122], which might have inflated estimates and increased the associations among variables. This, in turn, may limit the generalizability of our findings. Nevertheless, longitudinal studies are needed to disentangle the complex network of factors that may affect the professional quality of life of animal health-care providers. Furthermore, the data were collected entirely by self-report instruments, so the accuracy of individual reports cannot be guaranteed. Further research using a multimethod assessment of exposure to animal suffering, attachment styles, and professional quality of life is warranted. Moreover, as the cutoff of the Italian version of the Professional Quality of Life Scale [104] has not been established, we cannot describe the prevalence of the three dimensions in Italian veterinarians. In addition, a relatively modest portion of the total variance was explained by the selected predictors, indicating the possibility that our findings had been affected by other potentially significant variables (e.g., problems with affect regulation, psychiatric symptoms, or current social support) not explored here. Finally, despite the detection of some sex differences, we used a forced-choice format (0 = female; 1 = male) for indicating gender, hence data about other genders were not collected. Future studies should take pains to include transgender veterinarians to understand better their professional quality of life. Notwithstanding these limitations, the wide sample size and the relatively low overlapping among measures, as indicated by correlation coefficients, as well as the use of multivariate analysis, which reduces the rate of type-I error, allow us to maintain that our findings have a sufficient level of credibility and robustness. Hence, this initial study provided new evidence about the role that attachment styles may play in the multidetermined processes underlying veterinarians’ professional quality of life.

## Conclusions

In conclusion, we found that gender, workload, on call availability, exposure to animal suffering, and fearful and preoccupied attachment styles were associated with lower levels of veterinarians’ overall professional quality of life. This suggests that work-related factors may combine with contextual and individual psychological features in promoting or reducing professional quality of life. Specifically, these findings highlighted that veterinarians with high levels of attachment anxiety and with a negative self-view tend to become personally distressed and emotionally overwhelmed when providing care to suffering animals and lose their job satisfaction as helpers. Whereas, secure-attached veterinarians tend to display high levels of professional quality of life and less emotional distress even when their workload is high. These findings might have relevant implications for mitigating compassion fatigue and burnout in animal care provider settings and for promoting further research into risk factors and areas for potential intervention. In fact, our findings suggest that attachment styles are an important variable to take into account for planning educational programs addressed to veterinarians dealing with distressing circumstances, and this could help counselors understand the factors underlying low professional quality of life in their veterinarian clients, thereby tailoring psychological interventions for them. For example, an attachment-informed intervention program for veterinarians could aim at promoting positive views of the self and others, instead of limiting the scope of the intervention by attempting to reduce veterinarians’ exposure to sources of distress (i.e., exposure to animal suffering), which are characteristic elements of the profession. This more comprehensive approach may prove critical for improving the veterinarians’ overall professional quality of life, allowing them to work with even more compassion but also with less fatigue.

## Supporting information

S1 Table Gender differences for all investigated variables. (DOCX)

S1 Dataset (SAV)
