# Supplementary material for: Exposure to animal suffering, adult attachment styles, and professional quality of life in a sample of Italian veterinarians
Source: PLoS One. 2020 Aug 27;15(8):e0237991. doi: 10.1371/journal.pone.0237991 (PMC7451658; doi:10.1371/journal.pone.0237991)
Supplement: S1 Table — (DOCX) [file pone.0237991.s001.docx]

**S 1** Gender differences for all investigated variables

|  |  |  |  |  |  | |  |  |
| --- | --- | --- | --- | --- | --- | --- | --- | --- |
|  |  | Males (n = 434) |  |  | Females (n = 1,011) | | *t*_(1,443)_ | 95% CI |
|  |  | M (SD) |  |  | M (SD) |  |  |  |
| Compassion Satisfaction |  | 28.84 (5.34) |  |  | 27.78 (5.14) | | -3.54*** | [-1.64, -0.47] |
|  |  |  |  |  |  | |  |  |
| Burnout |  | 22.47 (5.79) |  |  | 24.65 (5.66) | | 6.66*** | [1.53, 2.82] |
|  |  |  |  |  |  | |  |  |
| Compassion fatigue |  | 12.11 (4.16) |  |  | 14.52 (5.12) | | 8.67*** | [1.87, 2.96] |
|  |  |  |  |  |  | |  |  |
| Hours of work per week |  | 44.09 (14.22) |  |  | 39.01 (14.10) | | -6.26*** | [-6.67, -3.49] |
|  |  |  |  |  |  | |  |  |
| Exposure to animal suffering |  | 16.98 (12.40) |  |  | 14.94 (11.45) | | -2.91** | [-3.41, -0.67] |
| Secure attachment |  | 3.88 (2.12) |  |  | 3.66 (2.04) | | -1.85 | [-0.15, 0.06] |
| Fearful attachment |  | 2.34 (1.60) |  |  | 3.05 (2.04) | | 6.46*** | [0.50, 0.93] |
|  |  |  |  |  |  | |  |  |
| Preoccupied attachment |  | 2.09 (0.50) |  |  | 2.33 (1.53) | | 2.45* | [0.05, 0.43] |
|  |  |  |  |  |  | |  |  |
| Dismissing attachment |  | 2.09 (1.53) |  |  | 3.70 (2.22) | | -2.98** | [-0.63, -0.14] |
|  |  |  |  |  |  | |  |  |

⁎ *p* < .05; ⁎⁎ *p* < .01; *** *p* < .001
